# Antioxidant Activity of Yichun Blue Honeysuckle (YBHS) Berry Counteracts CCl_4_-Induced Toxicity in Liver Injury Model of Mice

**DOI:** 10.3390/antiox6030050

**Published:** 2017-06-30

**Authors:** Mian-Ying Wang, Madhuwanti Srinivasan, Subramanyam Dasari, Parnal Narvekar, Angela Lincy Prem Antony Samy, Venkata Satish Dontaraju, Lin Peng, Gary L. Anderson, Gnanasekar Munirathinam

**Affiliations:** 1 Department of Pathology, University of Illinois College of Medicine, Rockford, IL 61107, USA; mianwangy@yahoo.com (M.-Y.W.); linpeng123@yahoo.com (L.P.); garyla@uic.edu (G.L.A.); 2 Department of Biomedical Sciences, University of Illinois College of Medicine, 1601 Parkview Avenue, Rockford, IL 61107, USA; madhuwanti.s@gmail.com (M.S.); sdasari7@uic.edu (S.D.); pnarve2@uic.edu (P.N.); aprema2@uic.edu (A.L.P.A.S.); 3 Internal Medicine, Rockford Memorial Hospital, Rockford, IL 61103, USA; vdontaraju@mhemail.org

**Keywords:** Yichun Blue Honeysuckle berry, liver toxicity, ROS, antioxidants

## Abstract

Yichun Blue Honeysuckle (YBHS) is reported to have a broad range of health benefits including protection against a number of chronic diseases. The objective of our study was to determine whether YBHS exhibits antioxidant activity, and if so, determine how it provides protection against oxidative stress. Eight-week old mice (25 male and 25 female) were randomized into five groups (*n* = 10 per group). YBHS extract (at 6.25%, 12.5%, or 25%) was administrated via intra-gastric tube to mice at 0.1 mL/10 g body weight once daily for 7 days. On the 8th day, all animals except for the controls received 250 mg/kg of CCl_4_ through an intra-gastric tube. The animals were sacrificed 6 h after CCl_4_ administration. Liver samples obtained from these mice were analyzed for the levels of Thiobarbituric Acid Reactive Substances (TBARS) and glutathione and the activities of Superoxide Dismutase (SOD), Catalase (CAT), and Glutathione Peroxidase (GPx), using biochemical assay kits. Our results showed that YBHS indeed reduces lipid peroxidation, suggesting that YBHS decreases the Reactive Oxygen Species (ROS) levels. We also found that YBHS activated the endogenous antioxidant enzymes including superoxide dismutase, catalase, and glutathione peroxidase and its co-enzyme glutathione reductase. In addition, we showed that glutathione levels were increased by YBHS treatment. Furthermore, the 2,2-diphenyl-1-picrylhydrazyl (DPPH) assay revealed that YBHS has potent free radical scavenging activity. Based on the results from our study, we conclude that YBHS scavenges ROS by enhancing the activity of the endogenous antioxidant defense system activity for conferring liver protective effects.

## 1. Introduction

Reactive Oxygen Species (ROS) are chemically reactive molecules containing oxygen. ROS are formed continuously in the human body during aerobic metabolism. During the process of the electron transport chain, a small percentage of electrons are directly transferred to oxygen, the terminal electron acceptor in the series, leading to superoxide radical formation. In healthy cells, ROS levels are maintained by the endogenous antioxidant defense system. In fact, ROS perform various useful functions such as blood pressure regulation, immune protection, and signal transduction [1]. Problems arise when the levels of ROS increase in such a way that the endogenous antioxidant defense system is not able to counteract them. The elevated levels of ROS (oxidative stress) can damage macromolecules such as DNA, proteins, and lipids [2]. In humans, oxidative stress is observed in most pathologies such as cancer [3], Alzheimer’s disease [4], and myocardial infarction [5]. For the past few decades, nutraceuticals and functional foods have become popular due to their potential to reduce health risks. Berries of various families such as strawberry, blackberry, and blueberry have been shown to have antioxidant properties [6]. Various berries have been reported to be rich in antioxidant compounds with antiradical functions [7,8,9]. Berries are found to have phenolic compounds and ascorbic acid, which are responsible for the improvement of various health conditions. Recently, *Lonicera caerulea* L. berry extract was found to attenuate liver toxicity via antioxidant, anti-inflammatory, and anti-apoptotic pathways [10]. Apart from aerobic metabolism, other sources of ROS are pollution, exotoxins such as mercury, lead, and cadmium, anticancer drugs, anesthetics, analgesics, and endotoxins such as bacteria, yeast, and viruses. Other examples of exogenous sources include stress, allergens, cold, excessive exercise, and dietary factors [11].

When ROS are produced, antioxidant enzymes present in the body stabilize them to delay or inhibit the oxidative damage to the cells. ROS are detoxified by the liver (the site of metabolism) in two phases. In phase 1 metabolism, the toxins form free radicals and undergo reactions such as oxidation, reduction, and hydrolysis. The free radicals generated in phase 1, which cause the oxidative stress, are eliminated in phase 2 by antioxidant enzymes. Enzymatic antioxidants are a network of interacting antioxidant enzymes through which cells protect themselves against oxidative stress. These include Superoxide dismutase (SOD), Catalase (CAT), Glutathione Peroxidase (GPx), and Glutathione Reductase (GR), which work synergistically together to detoxify free radicals [12]. The pathway starts with the enzyme SOD catalyzing the conversion of superoxide formed during the Electron Transport Chain pathway to hydrogen peroxide (H_2_O_2_) and oxygen [13,14]. Depending on their metal co-factor, SODs are classified into three families, namely, Cu/ZnSODs (bind to copper and zinc), FeSODs (bind to iron), and MnSODs (bind to manganese). Cu/ZnSODs are found in the cytoplasm while MnSODs are found in mitochondria [12]. CAT and GPx play a major role in neutralizing H_2_O_2_ [15,16]. Glutathione, a co-factor of GPx, is an endogenous antioxidant with a high antioxidant property and maintains the redox state of the cell [16]. Despite the innate antioxidant defense mechanism, oxidative stress may occur due to reasons such as mutations or inappropriate activation of ROS [17].

The damaging effects of ROS can be prevented by consuming food and other dietary sources rich in non-enzymatic antioxidants. These antioxidants include Vitamin C [18], Vitamin E [19,20,21], carotenoids, polyphenols, etc. [22]. Yichun Blue Honeysuckle (YBHS) is a less known fruit species which is now receiving attention for its health promoting substances including antioxidants, antimutagens, and anticarcinogens. This edible berry belonging to the species *Lonicera caerulae L. edulis* is grown in North-East China and has been used for its nutritional and medicinal values for over 3000 years. The YBHS used in this study is reported to contain anthocyanins, triterponic acids, β-carotene, ascorbic acid, catechol, flavonols, cholorogenic acid, and other acids, all of which are associated with high antioxidant activity. Among the anthocyanins, cyanidin-3-glucoside (C3G) is most dominant among this species. It is worth noting that C3G is generally considered to be the most active anthocyanin found in berries, particularly in the blue honeysuckle berry. Apart from cyanidin-3-glucoside, the other anthocyanins present in this species of BHS are cyanidin-3-rutinoside, cyanidin-3,5-diglucoside, malvidin-3-glucoside, and cyanidin-3-gentiobiosid. Many studies have shown that the antioxidant activity of *Lonicera caerulae* L. correlates with the amount of anthocyanin and polyphenols present [23].

This project aims to determine whether YBHS has protective effects against oxidative damage in a CCl_4_-induced liver injury model. In this study, we have evaluated the effects of YBHS on the lipid peroxidation and activity of endogenous antioxidant enzymes. 

## 2. Materials and Methods 

### 2.1. Materials

Sundried, wild blue honeysuckle berries were collected from the Xiaoxinganling mountain area and were provided by Heilongjiang Lannuo Health Food & Technology (Harbin, Heilongjiang, China). The assay kits for experiments such as the Thiobarbituric Acid Reactive Substances (TBARS) assay, SOD, CAT, and GPx activity assays, and Glutathione assay were from Cayman Chemical Company (Ann Arbor, MI, USA).

### 2.2. Methods

#### 2.2.1. Sample Preparation

Preparation of the water extract of blue honeysuckle berries: 20 grams of sun-dried wild blue honeysuckle berries were mixed with 200 mL acidic distilled water (200 mL) in a glass container and incubated at 4 °C for 24 h. The homogenized mixture was centrifuged at 600 g for 20 min. The supernatant was collected and filtered with Whatman No. 2 filter paper [24]. The water extract was collected for our experiments. CCl_4_-induced liver injury in mice was selected as a positive model, since CCl_4_ can be activated in vivo to form CCl_3_• free radicals causing lipid peroxidation to generate malondialdehyde (MDA) [25,26,27]. Six-week-old female and male ICR (Imprinting Control Region) mice were purchased from Charles River Inc. (Wilmington, MA, USA). The animals were housed in a room maintained at 25 °C with a 12 h photoperiod. They were fed a laboratory chow diet and water ad libitum. Animal work performed in this study was approved by the University of Illinois Rockford IACUC (Trial# 439-12). At eight weeks of age, the animals were divided into five groups of 10 mice each. Control animals were maintained on the regular diet and water while YBHS extract (25%, 12.5%, and 6.25%) were administrated to the experimental group at 0.1 mL/10 g body weight of the mouse per day for 7 days by intra-gastric tube. On the 8th day, all animals except for the controls received 250 mg/kg of CCl_4_ in corn oil through an intra-gastric tube. All animals were sacrificed 6 h post CCl_4_ administration and the liver tissues were removed and stored at −80 °C. For the antioxidant activity assays, we used the liver homogenates. Liver samples for various antioxidant assays were prepared as described previously with some modifications [28]. Briefly, the tissue was homogenized with HEPES buffer (1:5 dilutions) as required. Then the homogenized samples were centrifuged at 600 g, 4 °C for 15 min and the supernatants were collected. The supernatants were further centrifuged in the same conditions for 10 min at 9300 g to obtain the cytosol fraction and the mitochondrial fractions, respectively. 

#### 2.2.2. TBARS Assay

In order to determine whether YBHS decreases TBARS in the CCl_4_-induced liver injury model, we performed the TBARS assay using the kit from Cayman Chemical, Ann Arbor, MI, USA (catalogue no. 10009050). The level of MDA that is generated through lipid peroxidation, an indicator of oxidative stress in cells/tissues, is measured by the TBARS assay. This assay only provides a crude measure of lipid peroxidation. Thiobarbituric acid (TBA) provided in the kit combined with malondialdehyde (MDA) to form the MDA-TBA adduct (Thiobarbituric acid reactive substances) which can be detected colorimetrically. The assay was performed on the five groups of lysate samples obtained from the liver tissue treated with normal diet (control); normal diet and CCl_4_; normal diet and 25% YBHS + CCl_4_; normal diet and 12.5% YBHS + CCl_4_ group; and 6.25% YBHS + CCl_4_. The absorbance was measured at 530–540 nm. 

#### 2.2.3. SOD Assay

The impact of YBHS on the endogenous antioxidant enzymes, namely SOD, CAT, GPx, and Glutathione, was determined using the respective assay kits. Additionally, we also determined the impact of YBHS on Glutathione Reductase (GR), a co-enzyme of GPx. SOD plays a crucial cellular role in the antioxidant defense mechanism by catalyzing the dismutation of the superoxide anion to molecular oxygen and hydrogen peroxide. The Cayman Chemical superoxide dismutase assay kit (catalogue no. 706002) utilizes a tetrazolium salt for the detection of superoxide radicals generated by xanthine oxidase and hypoxanthine. The supernatant and mitochondrial lysates of liver tissues from the 5 groups of mice treated with: normal diet (control); normal diet and CCl_4_; normal diet and 25% YBHS + CCl_4_; normal diet and 12.5% YBHS + CCl_4_ group; and 6.25% YBHS + CCl_4_ were used to perform the assay following the protocol. Absorbance was measured at 440–460 nm using a plate reader. 

#### 2.2.4. CAT Assay

This endogenous antioxidant enzyme detoxifies the free radical hydrogen peroxide, which is toxic to cells. The Cayman Chemical catalase assay (catalogue no. 707002) method works based on the reaction of the enzyme with methanol in the presence of an optimal concentration of H_2_O_2_. The formaldehyde produced during the above reaction is measured colorimetrically using chromagen purpald. The steps to perform the assay were followed on the five sample groups of the CCl_4_-induced liver injury supernatant homogenate as per the manual in the kit. The absorbance was read at 540 nm using a plate reader. 

#### 2.2.5. GPx Assay

This enzyme protects cells from the oxidative stress reduction of hydrogen peroxides using reduced glutatiothione. The Cayman Chemical GPx assay kit (catalogue no.703102) determines the GPx activity by using cumene hydroperoxide as a substrate. The activity of GPx is measured indirectly by a coupled reaction with GR. The oxidation of NADPH to NADP^+^ is accompanied by a decrease in absorbance at 340 nm, which is proportional to the GPx activity. CAT assay samples were also used for the GPx assay.

#### 2.2.6. Glutathione Assay 

Cayman Chemical glutathione assay kit (catalogue no. 703002) provides the quantification of the total glutathione, i.e., GSH (reduced glutathione) and GSSG (oxidized glutathione). The assay utilizes the enzymatic recycling method, using glutathione reductase in order to measure the GSH. 

GSH reacts with DTNB (5,5’-dithio-bis-2-(nitrobenzoic acid) to produce a yellow colored TNB (5-thio-2-nitrobenzoic acid). The rate of TNB production is directly proportional to the formation of GSH which is measured at 405–414 nm using a plate reader at 5-min intervals for 30 min. The assay was performed on the supernatant, cytosolic, and mitochondrial liver homogenates of the five groups (control, CCl_4_, 25% BHS + CCl_4_, 12.5% BHS + CCl_4_, and 6.25% BHS + CCl_4_) to determine the effect of YBHS on glutathione levels in the respective samples.

#### 2.2.7. In Vitro Antioxidant Activity by DPPH Assay

The antioxidant activity of the YBHS water extract was assessed based on the DPPH (2,2-diphenyl-1-picrylhydrazyl) free radical method [29]. Briefly, various concentrations of YBHS sample extract in 1 mL were added to 3 mL ethanol containing 0.5 mM DPPH. When the DPPH free radical reacts with hydrogen-donating antioxidant compound, DPPH is reduced. The reduction was identified by the change in color from deep violet to light yellow, which was read at 517 nm. The mixture of ethanol (3.0 mL) and sample (1 mL) was used as a blank and the DPPH alone in ethanol was used as the control. Ascorbic acid was used as a positive control for the antioxidant activity. The percentage of scavenging activity was determined as described previously [29].

### 2.3. Statistical Analysis

Statistical analysis was performed with Graph Pad Prism 5 software (GraphPad Software, Inc., La Jolla, San Diego, CA USA). Data were compared between CCl_4_ and control groups, as well as between the CCl_4_ group and CCl_4_ plus 25% BHS, CCl_4_ group and CCl_4_ plus 12.5% BHS, and the CCl_4_ group and CCl_4_ plus 6.25% BHS group, respectively, by using Student’s *t*-test. *p* < 0.05 was considered statistically significant.

## 3. Results

### 3.1. YBHS Decreases Lipid Peroxidation in CCl_4_-Induced Liver Injury Model

**Figure 1:**
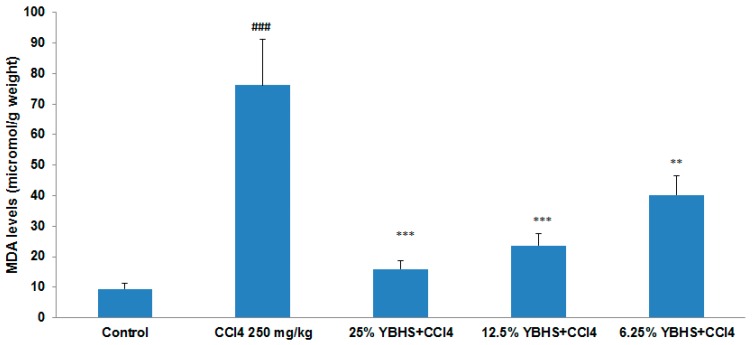
Impact of Yichun Blue Honeysuckle (YBHS) extracts on the malondialdehyde (MDA) levels of livers in different groups of mice. Based on the hypothesis that since YBHS have high anthocyanin content, it should have antioxidant activity, and the effects of YBHS water extract were studied on lipid peroxidation. In order to determine the antioxidant activity of YBHS, the Thiobarbituric Acid Reactive Substances (TBARS) assay was performed using homogenized liver samples of control, CCl_4_, CCl_4_ + 25% YBHS, CCl_4_ + 12.5% YBHS, and CCl_4_ + 6.25% YBHS. MDA levels in the CCl_4_ group were significantly increased compared to the control (### *p* < 0.001 highly significant compared to the control). YBHS extract was able to lower MDA with a dose response relationship in the YBHS extract treated groups compared with the CCl_4_ group (** *p* < 0.001 very significant, and *** *p* < 0.001 highly significant compared to CCl_4_). This data indicated that YBHS is efficient in reducing lipid peroxidation. The 25% YBHS treatment particularly showed a greater antioxidant effect. Data are represented as mean ± SD.

In order to determine the antioxidant activity of YBHS, the TBARS assay was performed using a kit obtained from Cayman Chemical. The homogenized liver samples of control, CCl_4_, CCl_4_ plus 25% BHS, CCl_4_ plus 12.5% BHS, and CCl_4_ plus 6.25% BHS groups were used. The results of the TBARS assay (Figure 1) indicated that the MDA levels in the three groups with the YBHS treatment were significantly decreased in comparison with the CCl_4_ group, which had an eight-fold increased MDA level compared to the control. Our results showed no statistical difference in the extent of damage/protection between female and male mice used in this study. The decrease in MDA levels suggests that YBHS is capable of reducing lipid peroxidation. 

### 3.2. YBHS Activates the Endogenous Antioxidant System 

**Figure 2:**
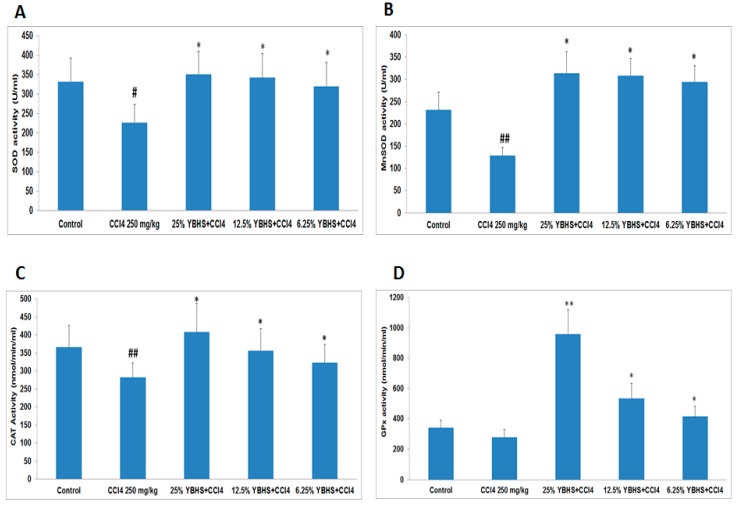
Impact of YBHS on total Superoxide Dismutase (SOD), Manganese Superoxide Dismutase (MnSOD), Catalase (CAT), and Glutathione Peroxidase (GPx) activity in the liver homogenate of ICR mice. (**A**) SOD activity was significantly reduced by the CCl_4_ treatment (CCl_4_ group). Results showed that YBHS extract was able to activate the SOD activity in all YBHS extract treated groups, namely, 25% YBHS + CCl_4_, 12.5% YBHS + CCl_4_, and 6.25% YBHS + CCl_4_ groups, respectively (# *p* < 0.05 significant compared to control; * *p* < 0.05 significant compared to CCl_4_). The 25% YBHS treatment group showed high SOD activity though the difference is not that significant between the three YBHS treatment groups; (**B**) YBHS extract increased mitochondria SOD (MnSOD) activity in different groups whereas SOD activity was significantly reduced by CCl_4_ treatment (## *p* < 0.01 very significant compared to the control). YBHS extract was able to activate SOD activity in 25% YBHS + CCl_4_, 12.5% YBHS + CCl_4_, and 6.25% YBHS + CCl_4_ groups significantly (* *p* < 0.05 significant, compared to CCl_4_); (**C**) CAT activity was significantly reduced by CCl_4_ treatment (in CCl_4_ group) (## *p* < 0.01 very significant compared to the control). On the other hand, the YBHS extract significantly activated the CAT activity in the 25% YBHS + CCl_4_, 12.5% YBHS + CCl_4_, and 6.25% YBHS + CCl_4_ groups (* *p* < 0.05 significant compared to CCl_4_). The highest CAT activity was observed in the 25% YBHS treatment group; (**D**) GPx activity was observed to be reduced by CCl_4_ treatment (in CCl_4_ group). YBHS water extract significantly activated the GPx activity in 25% YBHS + CCl_4_, 12.5% YBHS + CCl_4_, and 6.25% YBHS + CCl_4_ groups (** *p* < 0.01 very significant, * *p* < 0.05 significant compared to the CCl_4_ group). The 25% YBHS treatment group showed high GPx activity compared to the other two YBHS treatment groups. Data are represented as mean ± SD.

**Figure 3:**
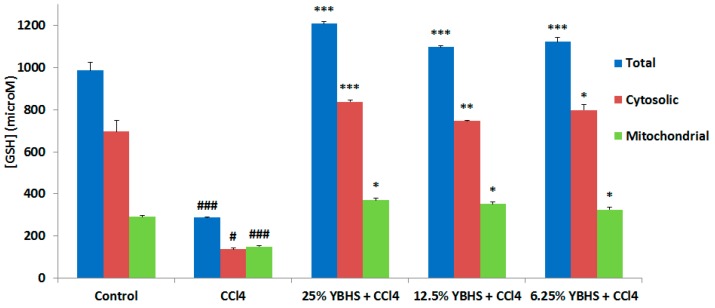
Impact of YBHS on the total (mitochondrial + cytosolic) glutathione (GSH) concentrations in the liver in different groups of mice. In order to confirm our hypothesis that YBHS may increase glutathione levels, the impact of YBHS on total, cytosolic, and mitochondrial samples of homogenized liver samples of the 5 different treatment groups indicated were studied using the Cayman Chemical glutathione assay kit. Glutathione levels were observed to be significantly reduced with CCl_4_ treatment compared to the control (# *p* < 0.05 significant, ### *p* < 0.001 extremely significant compared to the control) in all three fractions of the liver homogenate. YBHS treatment significantly increased (GSH) levels compared to the CCl_4_ group in the 25% YBHS + CCl_4_, 12.5% YBHS + CCl_4_, and 6.25% YBHS + CCl_4_ groups (* *p* < 0.05 significant, ** *p* < 0.01 very significant, *** *p* < 0.001 extremely significant compared to CCl_4_ group). Data are represented as mean ± SD.

In order to test our hypothesis, i.e., if the YBHS water extract enhances the endogenous antioxidant defense system activity, we tested the impact of YBHS on the endogenous antioxidant enzyme activity, namely, SOD, CAT, and GPx, using the respective kits from Cayman Chemical. We were also interested to see whether the YBHS treatment modulates the glutathione levels in mice. Again, we used the homogenized liver tissue samples of the five treatment groups. SOD converts the free radical O2•^−^ into H_2_O_2_, while CAT and GPx converts H_2_O_2_ into water. GPx requires secondary enzymes such as glutathione reductase (GR), and co-factors including Glutathione (GSH) and NADPH for high efficiency. Since these enzymes work synergistically, we were interested to see the impact of YBHS on the five antioxidant enzymes. For SOD, we used both supernatant (Cu/Zn SOD) and mitochondrial (MnSOD) tissue homogenates while we used only supernatant samples for CAT, GPx, and GR. Our results showed that YBHS increases the activity of the four antioxidant enzymes (Figure 2A–D) and GSH (Figure 3), comprising the endogenous antioxidant system compared to the CCl_4_ group. Thus, we can conclude that YBHS scavenges ROS by increasing the activity of the endogenous antioxidant system.

### 3.3. Free Radical Scavenging Activity of YBHS

**Figure 4:**
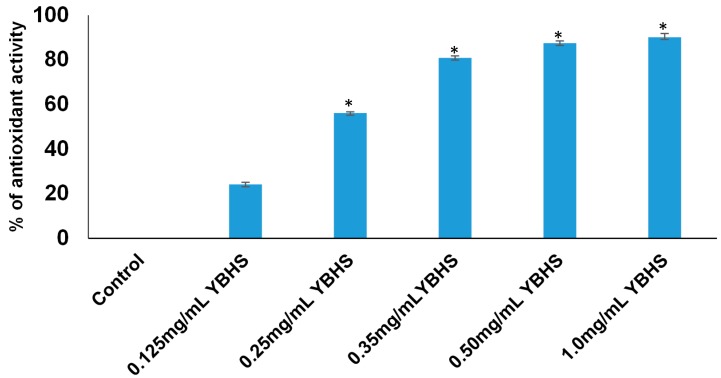
Antioxidant activity of the YBHS aqueous extract. The antioxidant/free radical scavenging activity of YBHS was determined by DPPH (2,2-diphenyl-1-picrylhydrazyl) assay. YBHS aqueous extract (0.25–1.0 mg/mL) shows significant (* *p* < 0.01) antioxidant activity compared to the control. The YBHS dose that required scavenging of 50% of the DPPH radical was found to be 0.25 mg/mL. Data are represented as mean ± SD.

The DPPH free radical assay is a simple and reliable method to study the antioxidant activity of plant extracts. As shown in Figure 4, aqueous extract of YBHS exhibited powerful DPPH radical scavenging activity (90.09% at 1.0 mg/mL of YBHS extract). The antioxidant activity of YBHS was evident even at a 0.125 mg/mL dose. Our data also showed that the YBHS extract dose-dependently increased the DPPH radical scavenging. 

## 4. Discussion

Oxidative stress is caused by Reactive Oxygen Species (ROS) that are formed constantly during aerobic metabolism. It is reported to be associated with about 200 diseases including cancer, diabetes, and cardiovascular and neurodegenerative diseases [30,31,32,33]. Reduction in the formation of ROS may be beneficial in preventing these chronic diseases. It is a well-known fact that small berries are rich in ROS scavenging antioxidants [34]. Among the less known edible small berries, YBHS shows promise as a remedy for oxidative stress. Though these berries have been used in folk medicine for many decades, it has only been recently suggested that the polyphenolic profile of BHS is responsible for the health benefits. The fruits are rich in antioxidants such as phenolic acids, flavonoids, anthocyanins, and proanthocyanidins. These aforementioned compounds, depending on their concentrations, may exert either positive or negative effects on various biological activities. YBHS may be a potential source for antioxidants to effectively overcome oxidative stress at physiologically or therapeutically relevant doses.

In order to study the antioxidant capability of YBHS, we employed a CCl_4_-induced mice liver injury model, which is a well-established model to study liver injury and oxidative damage [35]. In this study, we set out to study the effect of YBHS on lipid peroxidation by performing the TBARS assay which detects the levels of MDA, a by-product of lipid peroxidation. The assay with the liver homogenates of the five groups indicated that all three groups with the YBHS treatment decreased the MDA levels significantly in comparison with the CCl_4_ group, which suggests that YBHS is capable of reducing lipid peroxidation in a dose response relationship, with the 25% YBHS treatment showing the greatest effect. The mechanism by which YBHS reduced the levels of MDA is not known at this time. However, these findings correspond to the results of a similar TBARS study conducted on rat hepatocytes, where lipid peroxidation induced by tert-butyl hydroperoxide in the rat liver microsomes was reduced by phenolic fractions of BHS (*Lonicera caerulea* L.) [36]. Yet another study [37] conducted to determine the effects of 25% methanolic extracts of 12 cultivars of *Lonicera caerulea* L. on mice liver slice model peroxidation further confirmed that YBHS has the potential to decrease oxidative stress. 

We have demonstrated in our study that YBHS counteracts oxidative stress by activating endogenous antioxidant enzymes. The liver homogenate of the 25% YBHS treatment shows the greatest effect on MnSOD, CAT, and GPx activity. MnSOD is the key mitochondrial antioxidant enzyme which detoxifies the superoxide generated in mitochondria during oxidative stress [38]. Our data (Figure 2B) suggest that YBHS protects and enhances the MnSOD activity to overcome CCl_4_-mediated mitochondrial oxidative damage. Of note, GPx activity was found to be elevated in the YBHS treated groups (Figure 2D). The inactivation of GPx by physiological substances such as nitric oxide and carbonyl compounds leads to oxidative stress [39]. Given that GPx is the vital peroxide scavenging antioxidant enzyme present in the cell, our findings of YBHS enhancing GPx activity may have therapeutic relevance in pathologies associated with oxidative stress. In support of our study, a recent work by Wang et al. [10] also demonstrated the antioxidant activity of the *Lonicera caerulea* berry extract. However, their study showed the antioxidant potential of *Lonicera caerulea* on lipopolysaccharide-induced toxicity in a rat liver cell (BRL-3A) in vitro model. A previous study conducted using blueberries and strawberries also suggested that due to their polyphenolic profile, they boost endogenous antioxidant enzymes [40]. Another study proved the antiulcerogenic effect of *Solanum nigrum* berries (SBE) on aspirin-induced ulcerations in rat gastric mucosa by retaining SOD, CAT, GPx, and GSH activity at a physiological level when compared to the aspirin treated group [41]. Various berries have also been shown to scavenge free radicals [7,8,9]. In agreement with these studies, our findings showed that YBHS has the antioxidant capacity to scavenge DPPH free radicals. Thus, our study confirms that YBHS berries can activate all of the abovementioned endogenous antioxidant enzymes while increasing the endogenous antioxidant GSH, as well as scavenging free radicals. 

## 5. Conclusions

Taken together, our study suggests that YBHS is a strong antioxidant able to scavenge CCl_4_-induced MDA in liver. Furthermore, YBHS also fights oxidative stress by activating antioxidant enzymes, and also increases glutathione levels. It is worth noting that YBHS activated mitochondria function in the liver since the mitochondria MnSOD activity was selectively elevated in the YBHS pretreatment. YBHS may have a much greater antioxidant capacity than common known antioxidants such as vitamin C and E. More studies should be focused on the impact of YBHS on mitochondria function.

## References

[1] Devasagayam T.P., Tilak J.C., Boloor K.K., Sane K.S., Ghaskadbi S.S., Lele R.D. (2004). Free radicals and antioxidants in human health: Current status and future prospects. J. Assoc. Physicians India.

[2] Cheeseman K.H., Slater T.F. (1993). An introduction to free radical biochemistry. Br. Med. Bull..

[3] Halliwell B. (2007). Oxidative stress and cancer: Have we moved forward?. Biochem. J..

[4] Pohanka M. (2014). Alzheimer’s disease and oxidative stress: A review. Curr. Med. Chem..

[5] Dean O.M., van den Buuse M., Berk M., Copolov D.L., Mavros C., Bush A.I. (2011). N-acetyl cysteine restores brain glutathione loss in combined 2-cyclohexene-1-one and d-amphetamine-treated rats: Relevance to schizophrenia and bipolar disorder. Neurosci. Lett..

[6] Skrovankova S., Sumczynski D., Mlcek J., Jurikova T., Sochor J. (2015). Bioactive compounds and antioxidant activity in different types of berries. Int. J. Mol. Sci..

[7] Bae H.S., Kim H.J., Kang J.H., Kudo R., Hosoya T., Kumazawa S., Jun M., Kim O.Y., Ahn M.R. (2015). Anthocyanin profile and antioxidant activity of various berries cultivated in korea. Nat. Prod. Commun..

[8] Huang W.Y., Zhang H.C., Liu W.X., Li C.Y. (2012). Survey of antioxidant capacity and phenolic composition of blueberry, blackberry, and strawberry in Nanjing. J. Zhejiang Univ. Sci. B.

[9] Namiesnik J., Vearasilp K., Nemirovski A., Leontowicz H., Leontowicz M., Pasko P., Martinez-Ayala A.L., Gonzalez-Aguilar G.A., Suhaj M., Gorinstein S. (2014). In vitro studies on the relationship between the antioxidant activities of some berry extracts and their binding properties to serum albumin. Appl. Biochem. Biotechnol..

[10] Wang Y.H., Li B., Lin Y., Ma Y., Zhang Q., Meng X.J. (2017). Effects of *Lonicera caerulea* berry extract on lipopolysaccharide-induced toxicity in rat liver cells: Antioxidant, anti-inflammatory, and anti-apoptotic activities. J. Funct. Foods.

[11] Cadenas E., Davies K.J. (2000). Mitochondrial free radical generation, oxidative stress, and aging. Free Radic. Biol. Med..

[12] Lobo V., Patil A., Phatak A., Chandra N. (2010). Free radicals, antioxidants and functional foods: Impact on human health. Pharmacogn. Rev..

[13] Zelko I.N., Mariani T.J., Folz R.J. (2002). Superoxide dismutase multigene family: A comparison of the CuZn-SOD (SOD1), Mn-SOD (SOD2), and EC-SOD (SOD3) gene structures, evolution, and expression. Free Radic. Biol. Med..

[14] Bannister J.V., Bannister W.H., Rotilio G. (1987). Aspects of the structure, function, and applications of superoxide dismutase. CRC Crit. Rev. Biochem..

[15] Chelikani P., Fita I., Loewen P.C. (2004). Diversity of structures and properties among catalases. Cell. Mol. Life Sci..

[16] Meister A., Anderson M.E. (1983). Glutathione. Annu. Rev. Biochem..

[17] Kohen R., Nyska A. (2002). Oxidation of biological systems: Oxidative stress phenomena, antioxidants, redox reactions, and methods for their quantification. Toxicol. Pathol..

[18] Padayatty S.J., Katz A., Wang Y., Eck P., Kwon O., Lee J.H., Chen S., Corpe C., Dutta A., Dutta S.K. (2003). Vitamin C as an antioxidant: Evaluation of its role in disease prevention. J. Am. Coll. Nutr..

[19] Herrera E., Barbas C. (2001). Vitamin E: Action, metabolism and perspectives. J. Physiol. Biochem..

[20] Brigelius-Flohe R., Traber M.G. (1999). Vitamin E: Function and metabolism. FASEB J..

[21] Traber M.G., Atkinson J. (2007). Vitamin E, antioxidant and nothing more. Free Radic. Biol. Med..

[22] Rice-Evans C.A., Miller N.J., Paganga G. (1996). Structure-antioxidant activity relationships of flavonoids and phenolic acids. Free Radic. Biol. Med..

[23] Jurikova T., Rop O., Mlcek J., Sochor J., Balla S., Szekeres L., Hegedusova A., Hubalek J., Adam V., Kizek R. (2011). Phenolic profile of edible honeysuckle berries (genus *Lonicera*) and their biological effects. Molecules.

[24] Ningappa M.B., Dinesha R., Srinivas L. (2008). Antioxidant and free radical scavenging activities of polyphenol-enriched curry leaf (*Murraya koenigii* L.) extracts. Food Chem..

[25] Recknagel R.O. (1967). Carbon tetrachloride hepatotoxicity. Pharmacol. Rev..

[26] Liu S.L., Degli Esposti S., Yao T., Diehl A.M., Zern M.A. (1995). Vitamin E therapy of acute CCl_4_-induced hepatic injury in mice is associated with inhibition of nuclear factor kappa B binding. Hepatology.

[27] Wang M.Y., Nowicki D., Anderson G., Jensen J., West B. (2008). Liver protective effects of *Morinda citrifolia* (Noni). Plant Foods Hum. Nutr..

[28] Laouar A., Klibet F., Bourogaa E., Benamara A., Boumendjel A., Chefrour A., Messarah M. (2017). Potential antioxidant properties and hepatoprotective effects of *Juniperus phoenicea* berries against CCl_4_ induced hepatic damage in rats. Asian Pac. J. Trop. Med..

[29] Mensor L.L., Menezes F.S., Leitao G.G., Reis A.S., dos Santos T.C., Coube C.S., Leitao S.G. (2001). Screening of brazilian plant extracts for antioxidant activity by the use of DPPH free radical method. Phytother. Res..

[30] Droge W. (2002). Free radicals in the physiological control of cell function. Physiol. Rev..

[31] Kovanen P.T. (1993). The mast cell—A potential link between inflammation and cellular cholesterol deposition in atherogenesis. Eur. Heart J..

[32] Marnett L.J. (2000). Oxyradicals and DNA damage. Carcinogenesis.

[33] Shimoda R., Nagashima M., Sakamoto M., Yamaguchi N., Hirohashi S., Yokota J., Kasai H. (1994). Increased formation of oxidative DNA damage, 8-hydroxydeoxyguanosine, in human livers with chronic hepatitis. Cancer Res..

[34] Zafra-Stone S., Yasmin T., Bagchi M., Chatterjee A., Vinson J.A., Bagchi D. (2007). Berry anthocyanins as novel antioxidants in human health and disease prevention. Mol. Nutr. Food Res..

[35] Tomasi A., Albano E., Banni S., Botti B., Corongiu F., Dessi M.A., Iannone A., Vannini V., Dianzani M.U. (1987). Free-radical metabolism of carbon tetrachloride in rat liver mitochondria. A study of the mechanism of activation. Biochem. J..

[36] Palikova I., Valentova K., Oborna I., Ulrichova J. (2009). Protectivity of blue honeysuckle extract against oxidative human endothelial cells and rat hepatocyte damage. J. Agric. Food Chem..

[37] Rop O., Reznicek V., Mlcek J., Jurikova T., Balik J., Sochor J., Kramarova D. (2011). Antioxidant and radical oxygen species scavenging activities of 12 cultivars of blue honeysuckle fruit. Hortic. Sci..

[38] Bresciani G., da Cruz I.B., Gonzalez-Gallego J. (2015). Manganese superoxide dismutase and oxidative stress modulation. Adv. Clin. Chem..

[39] Miyamoto Y., Koh Y.H., Park Y.S., Fujiwara N., Sakiyama H., Misonou Y., Ookawara T., Suzuki K., Honke K., Taniguchi N. (2003). Oxidative stress caused by inactivation of glutathione peroxidase and adaptive responses. Biol. Chem..

[40] Poulose S.M., Bielinski D.F., Carrihill-Knoll K.L., Rabin B.M., Shukitt-Hale B. (2014). Protective effects of blueberry- and strawberry diets on neuronal stress following exposure to ^56^Fe particles. Brain Res..

[41] Jainu M., Devi C.S. (2004). Antioxidant effect of methanolic extract of *Solanum nigrum* berries on aspirin induced gastric mucosal injury. Indian J. Clin. Biochem..

